# Congestive Nephropathy

**DOI:** 10.3390/ijerph19052499

**Published:** 2022-02-22

**Authors:** Luis D’ Marco

**Affiliations:** Departamento de Medicina y Cirugía, Facultad Ciencias de la Salud, Universidad Cardenal Herrera-CEU, CEU Universities, 46115 Valencia, Spain; luisgerardodg@hotmail.com

**Keywords:** congestion, chronic kidney disease, heart failure, cardiovascular disease

## Abstract

The complex interaction between cardiac and renal functions is known. However, when these functions are disrupted, many intricate and sensitive interactions between these organs are failed by several pathophysiological ways. As a result, this malfunction is clinically evident by sign and symptoms associated to intravascular and interstitial congestion. In this sense, the adverse impact of venous congestion on renal function has long been recognized. Currently, the presence of a specific subtype of nephropathy associated to congestion has been suggested. Even though no diagnosis criteria has been clearly stablished, and no renal specific histological pattern were reported; studies regarding this issue may help to improve the handling and therapeutic principles in affected patients.

## 1. Introduction

The cardiorenal and vascular axis are necessary for cardiovascular homeostasis, and the maintenance of vitals function depends on many intricate and sensitive interactions between these organs [1]. This complex interaction is fine-tuned by many systems such as the renin–angiotensin–aldosterone system (RAAS), sympathetic nervous system (SNS), natriuretic peptides, maintenance of fluid and electrolytes, and acid–base equilibrium [2].

In recent years, the coexistence of cardiovascular and renal diseases has led to a proposal of cardiorenal syndromes defined as “a complex pathophysiological disorder of the heart and the kidneys, whereby acute or chronic dysfunction in one organ may induce acute or chronic dysfunction in the other organ” [3]. Hence, this classification provides a clinically oriented descriptive definition, however, poorly tested in clinical practice or in many clinical trials.

The term congestive nephropathy was raised recently to describe the neglected clinical entity of renal dysfunction associated with venous congestion as well as reduced renal perfusion observed mainly in those patients with cardiorenal compromise [4]. In this sense, this hemodynamic phenotype of renal dysfunction is still not clearly defined. One of the main reasons of the scarce evidence supporting this term is associated with the common comorbidities related to cardiorenal syndromes; thus, diabetes, hypertension, and other vascular diseases are present in affected patients at the time of diagnosis [5]. Moreover, there is no indication of kidney biopsy in patients with cardiorenal syndrome, since the clinicians assume that diabetic nephropathy and/or hypertensive nephrosclerosis are always involved [4,6].

This review explores the implications of this novel term called “congestive nephropathy” and some mechanisms that may explain the pathophysiology of this condition in affected patients. 

## 2. Pathophysiology of Congestive Nephropathy

Heart failure and chronic kidney disease are commonly associated conditions, and congestive nephropathy should be suspected in patients clinically affected who show an improvement in renal function after decongestion therapies. From a hemodynamic point of view, the increase in central venous pressure (CVP) is directly transmitted to the renal venous system and, consequently, influences the glomerular filtration rate (GFR) [7,8,9]. The renal filtration pressure (FP) (from which the hydrostatic pressure of the glomerular capillary comes) depends on the renal perfusion pressure (RPP) and the renal blood flow (RBF). In turn, RPP depends on mean arterial pressure (MAP) and renal venous pressure (RVP), the RBF of renal arterial pressure (RAP), RVP, and intrarenal vascular resistance (IVR) [4]. Of interest, an increase in CVP may be prevented by a compensatory increase in renal lymphatic flow, however, the continuing elevation of CVP will progressively reduce venous and lymphatic outflow and late-stage arterial inflow [10].

**Figure 1:**
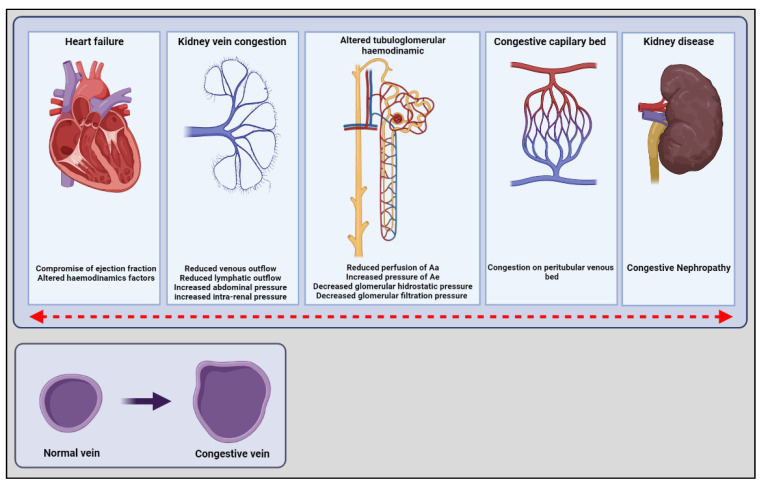
The settings of congestive nephropathy. The figure shown the cardiovascular pressure changes in a long-term scenario. These mechanisms can be associate to congestive nephropathy, mainly by kidney vein congestion, altered tubuloglomerular hemodynamics leading to an stablish disease. The red arrow expresses the bidirectional alterations of these changes. Additionally, the small figure shown the anatomical changes that volume overload and congestion cause in veins structure. Aa, arteriole afferent; Ae; arteriole efferent.

In the model of patients with type 2 cardiorenal syndrome with chronic heart failure, the RBF and GFR remain almost constant within a relatively wide range of RPP due to the mechanisms of autoregulation and tubuloglomerular feedback that modify pre- and post-glomerular resistance to maintain FP [9]. On the contrary, in the context of acute heart failure (type 1 cardiorenal syndrome), the self-regulatory mechanisms are altered, and the FP is highly dependent on the balance between MAP and PVR [11]. Therefore, the increase in PVR (intrarenal afterload) can significantly reduce the RBF. Likewise, since the kidney is a encapsulated organ, the increase in PVR produces mechanical compression on the interstitium and intratubular compartment, which further reduces the glomerular transcapillary hydrostatic pressure gradient and, therefore, represents an additional mechanism through which renal venous hypertension can compromise GFR [2]. Similarly, renal venous congestion produces inflammation, oxidative stress, and renal ischemia, producing intrinsic tubular damage [4,7,9]. Finally, in a long-term scenario, these mechanisms can be associate to congestive nephropathy, mainly as a tubular damage. Furthermore, volume overload is reported to be associated with the decline in renal function and disease progression (Figure 1).

The pathophysiology of congestive nephropathy in the setting of hemodynamic disfunction is complex and multifactorial. Beyond these pressure changes, the renal structural damage and other system alterations should explain many of the underlying intrinsic kidney changes. Thus, these changes remain an important determinant of the “kidney reserve” available to relieve congestion and to respond to the insult posed by cardiac disfunction and the aggressive diuresis and natriuresis necessary during the decongestion treatment.

As commented previously, this cardiorenal interaction is fine-tuned by neurohormonal activity, including RAAS hyperactivity, SNS over-reactivity, and natriuretic peptides [1]. In these regards, SNS, RAAS, non-osmotic vasopressin, and nitric oxide depletion are the most important mediators of intrarenal mechanisms of adaptation [12]. In this setting, other co-factors such as inflammatory cytokine release, oxidative stress, and endothelial dysfunction worsen the hemodynamic disorders and cooperate in further alterations of GFR. Eventually, acute renal dysfunctions or even acute tubular necrosis could occur, which might lead to a hypoxic state of the renal parenchyma, tubulo-interstitial fibrosis, and glomerulosclerosis resulting in worsening of renal function in chronic kidney disease patients, leading to end stage kidney disease, and could be long-term consequences [9]. Lastly, the histological pattern of congestive nephropathy could be a mixture between acute and chronic findings that depend in part on the evolution time of the disease. 

## 3. Diagnostic

Congestive nephropathy is closely associated to volume overload and cardiorenal compromise. Hence, this condition may be suspected in patients with acute or chronic cardiorenal dysfunction and clinical evidence of congestion. Moreover, the potentially reversible condition of this subtype of renal dysfunction has been suggested [4]. Commented earlier, the uses of adequate decongestion therapies could help to improve and reverse cardiorenal dysfunction. Nevertheless, the evolution time of the hemodynamic changes will lead to an acute associated renal variation or to a chronic structural and cellular damage. These final stages of the renal compromise observed in affected patients may be susceptible to an additional exploration such as histopathological lesion patterns that will guide clinicians in the course of the disease evolution.

Beyond the tentative benefits of the histologic changes and invasive methods to measure intravascular venous pressure, there are non-invasive techniques that may help in the diagnostic approach. Thus, computer tomography, magnetic resonance, and ultrasound imaging techniques have been studied in the evaluation of patients with congestion in cardiac and renal disfunction. Recently, a four-points ultrasound evaluation has been proposed with the aim to monitor the basal state and the evolution of the decongestive therapies [13,14]. The ultrasound spectral analysis wave patterns of the cava, hepatic, porta, and intra-renal veins are a useful approach in congestive patients. 

The assessment of volume status can reach by analytical biomarkers. Elevated levels of natriuretic peptides are indicators of cardiac and intravascular congestion [15]; however, they are influenced by some medication and renal dysfunction [16]. In this sense, natriuretic peptide results must always be interpreted in the context of clinical findings. More recently, they have tested the potential value of carbohydrate antigen (CA)- 125 in patients with heart failure [17]. Although it has traditionally been used for monitoring and risk stratification in ovarian cancer, elevated plasma levels of CA-125 have been identified in other entities related to hydropic states [18]. Although the pathophysiological mechanism linked to heart failure is not fully understood. One of the most accepted theories suggests the activation of mesothelial cells in response to increased hydrostatic pressure, mechanical stress, and inflammatory cytokines. This biomarker is released by epithelial serous cells (pericardium, pleura, and/or peritoneum) in response to mechanical (hydrostatic pressure) or cytokine stimuli [4,9,17,18]. On the contrary to natriuretic peptides, CA-125 production is not influenced by age or renal function [16]. Finally, the classic serum and urinary biomarkers of renal disfunction such as creatinine, urea, sodium, and potassium may be interpreted with caution in the context of other clinical and imaging methods to improve the diagnostic assessment of congestive nephropathy. 

## 4. Therapeutic Approaches

Currently, the benefits of the multidisciplinary handling of patients with congestion and cardiorenal syndrome has been reported [19,20]. Moreover, in many hospitals, these patients are treated early by a team involving cardiologists and nephrologists in cardiorenal units. In this sense, the main objective in the treatment of congestive nephropathy will be associate to counteract the major modifiable pathophysiological abnormalities [4,9]. Hence, one of the targets is to control the hemodynamic abnormalities, pressure homeostasis, and renal function. On the other hand, avoid the underuse or overuse of cardiovascular drugs and interventions in susceptible patients with previous moderate or severe chronic kidney disease [21]. In addition, it is necessary to differentiate the renal state before the initiation of an aggressive decongestion treatment [22].

Although an in-depth analysis of the current decongestion therapies is not the objective of this review, some changes have been made in recent years in this regard. As an initial measure, avoid congestion is reached with salt (and water in hyponatremia) restriction [21]. Diuretics are commonly used to treat fluid overload and renal congestion. Moreover, loop diuretics are the cornerstone of treatment. As is usual, the association of thiazide-like diuretics, such as metolazone and/or potassium-sparing diuretics, are often used to overcome the increased distal sodium reabsorption due to the chronic use of loop diuretics [2]. In the case of diuretic resistant with metabolic alkalosis, the association of acetazolamide to loop diuretics is particularly effective and the switch from furosemide to torasemide in resistant patients.

Hypothetically, congestion can also be reduced by increasing splanchnic vascular capacitance by ACE inhibitors and/or β-blockers [23]. Moreover, natriuretic peptides improve renal perfusion by reducing preglomerular vascular pressure and resistance, increase the filtration surface by relaxing mesangial cells, and stimulate diuresis and natriuresis by direct glomerular mechanisms (tubuloglomerular balance). However, its biological function is compromised in patients with heart failure due to degradation mediated by neprilysin activity. Therefore, sacubitril/valsartan combines the benefits derived from the inhibition of the RAAS with the reduction in the degradation of natriuretic peptides because of the inhibition of neprilysin [24].

More recently, sodium-glucose cotransporter type 2 (SGLT2) inhibitors have been consistently shown to improve congestion in affected patients regardless of ejection fraction [25]. These hypoglycemics drugs have a diuretic effect (through a reduction of proximal sodium reabsorption and osmotic diuresis) and paradoxically cause a reduction in RAAS hyperactivity [26,27].

## 5. Conclusions

At present, the presence of congestive nephropathy and diagnosis criteria has not been clearly stablished in the nephrology community. Furthermore, there is no specific histological pattern reported. However, the adverse impact of venous congestion on renal function has long been recognized. Finally, studies regarding this issue may help to improve the handling and therapeutic principles in the clinical practice.

## Data Availability

Not applicable.
